# Glutaric AciduriaType 1: Clinical and Molecular Study in Iranian Patients, 3 Novel Mutations

**Published:** 2017

**Authors:** Zahra PIRZADEH, Massoud HOUSHMAND, Jafar NASIRI, Mohsen MOLLAMOHAMMADI, Mostafa SEDIGHI, Seyed Hassan TONEKABONI

**Affiliations:** 1 Children Growth Research Center, Qazvin University of Medical Science, Qazvin, Iran.; 2 Department of Medical Genetics, National Institute for Genetic Engineering and Biotechnology, Tehran, Iran.; 3 Child Growth and Development Research Center, Isfahan University of Medical Sciences, Isfahan, Iran.; 4 Qom University of Medical Sciences, Qom, Iran.; 5 Kermanshah University of Medical Sciences, Kermanshah, Iran.; 6 Pediatric Neurology Research Center, Research Institute for Children Health, ShahidBeheshti University of Medical Science, Tehran, Iran; School of Medicine, ShahidBeheshti University of Medical Science, Tehran, Iran.

**Keywords:** Glutaricaciduria type1, Glutaryl co-A dehydrogenase, GCDH mutation, Iran

## Abstract

**Objective:**

Glutaricaciduria type 1 (GA1), is a rare, treatable neuro metabolic disease, due to *glutaryl- CoA dehydrogenase (GCDH)* gene mutation.In regions without neonatal blood screening (NBS), patients are diagnosed in symptomatic period. This study was carried out to assess patients with GA1 for clinical, biochemical, neuroimaging findings and GCDH gene mutations analysis.

**Materials & Methods:**

In this cross-sectional study, clinical manifestation, neuroimaging and metabolic findings of eleven Iranian GA1 patients of MofidChildren’s Hospital, Tehran, Iranbetween 2001 and 2011,were evaluated.Mutational analysis of the GCDH gene was performed on genomic DNA. Genomic DNA was extracted from peripheral lymphocytes using QIAamp DNA Micro Kit (Qiagen). All 11 exons and flanking intronic regions of the GCDH gene were amplified by polymerase chain reaction (PCR).

**Results:**

All patients were diagnosed before 32 months old. Clinical presentations of GA1 include acute encephalopathic crisis and/or developmental delay and macrocephaly. Seven GCDH gene mutations were detected in our patients. The most frequent GCDH mutations occurred in exon7 then exon8, 10 and11. G244 C in exon7, R294 Q in exon8 and N373 S in exon 10 were three novel mutations. There was no correlation between of genotype and phenotype in our patients.

**Conclusion:**

Physician must remember GA1 in differential diagnosis of acute encephalopathic crisis, macrocephaly, developmental delay, movement disorders such as dystonia and dyskinesia. Early detection, proper treatment and selective screening of patients’ siblings can prevent neurologic disabilities.

## Introduction

Glutaricaciduria type 1(GA1, #231670) was introduced as a new neurodegenerative disorder in 1975 (1). This progressive neurometabolic disorder with characteristic clinical presentation usually manifests with macrocephaly, acute encephalopathic crisis after upper respiratory/gastrointestinal infection,fever, dehydration, vaccination orin lesser extent with insidious-onsetpresentation such as movement disorder (dystonia, ataxia ), developmental delay(2-4).Occasionally child physical abuse has been suspected because of presence of subdural effusions (5). Life expectancy is greatly reduced in symptomatic patients with complex movement disorder (6).The peak age of presentation is 9 months and most symptomatic patients present before 3 yr of age; althoughrare symptomatic neurological presentation (late onset) are reported after 5 yr old of age(7,8). 

Frequent neuroimaging findings in GA1 are described in the different medical literature include widened Sylvianfissures, frontotemporal volume loss, ventriculomegaly, subdural hematomas, delayed myelination demyelination and basal ganglia lesions (9,10).

Deficient activity of mitochondrial enzyme glutaryl-CoA dehydrogenase (GCDH; EC.1.3.99.7) that is essential for degradation of lysine, hydroxylysine and tryptophan pathway is responsible for this autosomal recessive disorder of inborn error of metabolism. Thus, the biochemical diagnosis is based on detection of increased levels glutaric acid, 3-hydroxyglutaric acid in urine, other physiologic body fluid and elevation glutarylcarnitine (C5DC) level in plasma or urine (11). Suspected GA1 patients based on clinical, biochemical, neuroimaging finding is confirmed by *GCDH* gene mutations or GCDH enzyme activity.

*GCDH* gene encodes GCDH enzyme located on chromosome19p13.2and is composed of 11 exons and 10 introns (12, 13).More than 200 mutations have been reported in the *GCDH* gene (14). However,some *GCDH* gene mutations are frequent in specific population.

This study was carried out to assess patients with GA1 for clinical, biochemical, neuroimaging findings and *GCDH* gene mutations analysis.

## Materials&Methods

We studied 11Iranian patients with GA1 previously diagnosed in Neurology and Endocrinology Clinics of MofidChildren’s Hospital, Tehran, Iranbetween 2001 and 2011.Fourteen Iranian GA1 patients were diagnosed during these 10 yr.One patient died. One family did not accept to test their child for *GCDH* gene mutation. One patient’s family immigrated to another country. Diagnoses of these symptomatic patients were based on glutarylcarnitine analysis in their dried blood spots or urine and/or their urinary organic acid profiles. They were invited to hospital for interview, physical examination, collecting their urine and blood laboratory information, neuroimaging findings and performing *GCDH* gene mutation study. GCDH gene mutation studies were done in Special Medical Center, Tehran, Iran.

Mutational analysis of the *GCDH* gene was performed on genomic DNA. Genomic DNA was extracted from peripheral lymphocytes using QIAamp DNA Micro Kit (Qiagen). All 11 exons and flanking intronic regions of the *GCDH* gene were amplified by PCR. Primer sequences are given in Table 1 (13). PCR was performed in a total volume 25 µl containing1x PCR buffer,1.5mM MgCl2, 8 pmol each primer, 0.2 mM each dNTP, 50 ng template DNA, and 0.3 U Taq polymerase (SinaClon, Iran). Five percent DMSO was also added for PCR of exon 2.Amplification was performed with initial denaturation at 94ºC for 5 min,32 cycles of 1 min at 94ºC, 1 min at 55-62ºC atannealing according to Table 1, and 1 min at 72ºC and final extension were 5 min at 72ºC. The PCR products were sequenced on ABI 3700 sequencer (Kosar Company, Tehran).Sequencing results were compared with ref sequences in NCBI.

Informed consent to perform DNA analysis wasobtained from the parents of the patients. The Ethics Committee of the Faculty of Medicine,ShahidBeheshti University of Medical Sciences, Tehran, Iran approved our study protocol. 

## Results

The clinical and molecular findings of 11 Iranian GA1 patients are summarized in Table 1.

**Table 1:** Clinical and molecular findings in 11 Iranian patients with glutaricaciduria type 1

**Patient ID**	**Age atdiagnosismonths**	**Age at mutation analysis (months)**	**Presentation**	**Macrocephaly after birth**	**Exon**	**Nucleotide substitution**		**Amino-acid change**	**Mutation**	**Movement disability**	**Reference**
1	9	52	Developmental delay, dystonia,	No	7	C>T	CCC>CTC	Pro>Leu	P 248 L	severe	13
2	11	13	Developmental delay, dystonia,	No	7	”	”	”	”	moderate	”
3	7	44	Developmental delay, dystonia,	Yes	7	”	”	”		severe	”
4	32	34	Developmental delay,dystonia	No	7	T>C	TTC>CTC	Phe>Leu	F236L	severe	28
5	12	23	Acute: seizure	Yes	7	G>T	GGT>TGT	Gly>Cys	G244C	severe	
6	17	17	Acute:: postvaccinal seizure	No	11	C>T	ACG>ATG	Thr>Met	T429M	normal	30
7	30	59	Developmental delay, dystonia,	Yes	11	C>T	GCG>GTG	Ala>Val	A433V	severe	29
8	11	65	Acute:stausepilepticus,	Yes	10	A>G	AAT>AGT	Asn>Ser	N373S	mild	
9	11	65	Acute: neonatal and postvaccinal seizure,	Yes	10	”	”	”	”	mild	
10	5	15	Acute:irritability encephalopathy-	Yes	8	G>C	CGG>CCG	Arg>Pro	A294P	mild	
11	15	102	Acute:irritability encephalopathy-	Yes	8	”	”	”	”	severe	

**Demographic and clinical findings**

**Fig1:**
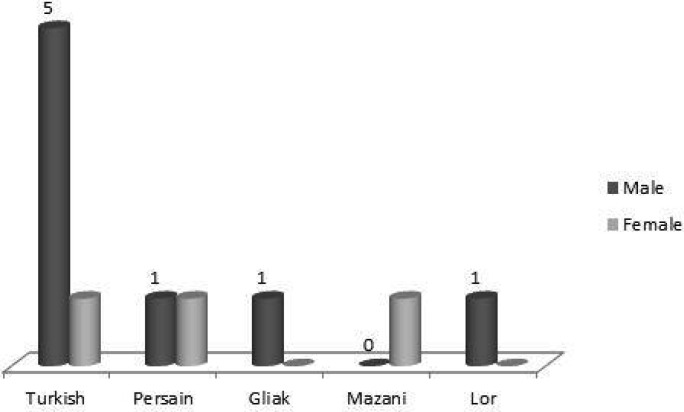
Frequency of patients with GA1 according to gender and ethnicity

Eleven symptomatic Iranian patients with GA1 were diagnosed from 10 families. The only sibling patients were two brothers, delivered as triple pregnancy. All of patients were offspring of consanguineous marriages.Ethnic origin and gender frequency ofour patients are shown in Figure 1.

The mean age at diagnosis was 14.8 (range: 5-32) months. The mean age at study time was 44.5 (range 15-102) months. Acute encephalopathic crisis and seizure following URI or vaccination were the first clinical presentations in six cases. Macrocephaly, developmental delay and extrapyramidal signs were the first presentations in other five patients. Birth head circumferences of all patients were under percentile 95(percentile25-90).Patients were treated by low protein (lysine,tryptophan,etc.) diets and carnitine supplementation. Treatments were discontinued in five patients because their families did not fill any improvement in their child symptoms and signs. Other families tried to continue this treatment, although these patients were diagnosed, recently

**Neuroimaging findings**

**Fig 2:**
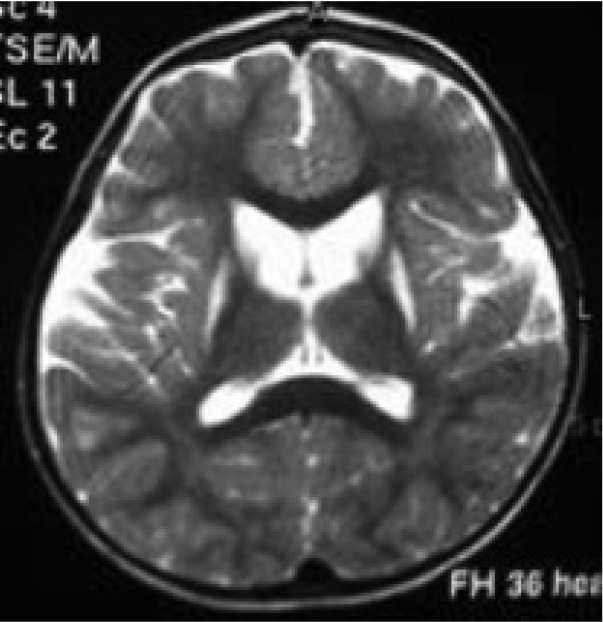
Axial T2 weighted MRI scan of patient 11 shows basal ganglia (putamen) hyperintensities

**Fig 3:**
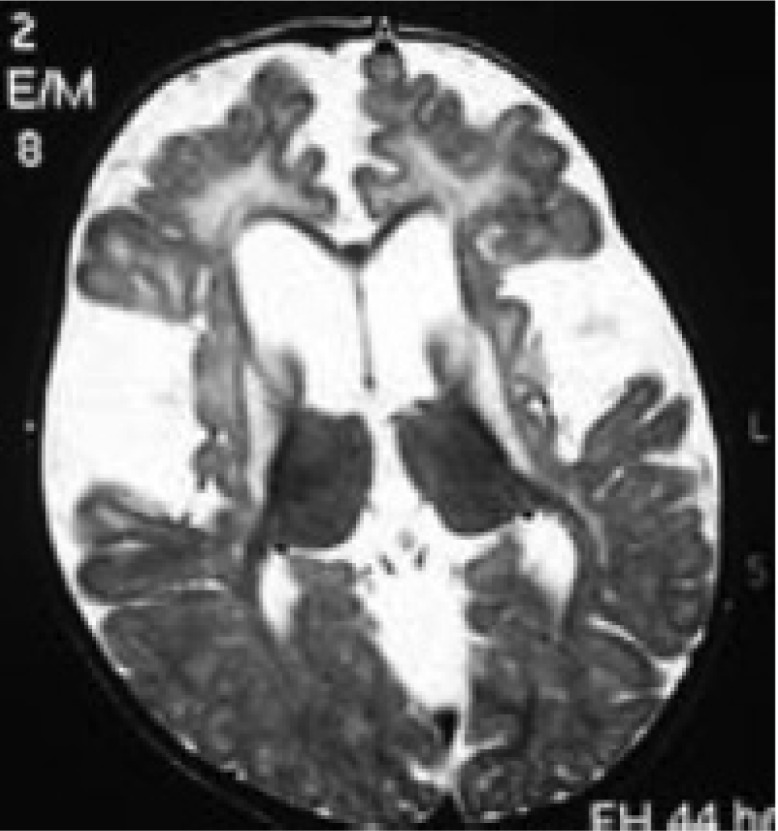
Axial T2 weighted MRI scan of patient 4 shows frontotemporal brain atrophy, temporal hypoplasia,

**Fig 4:**
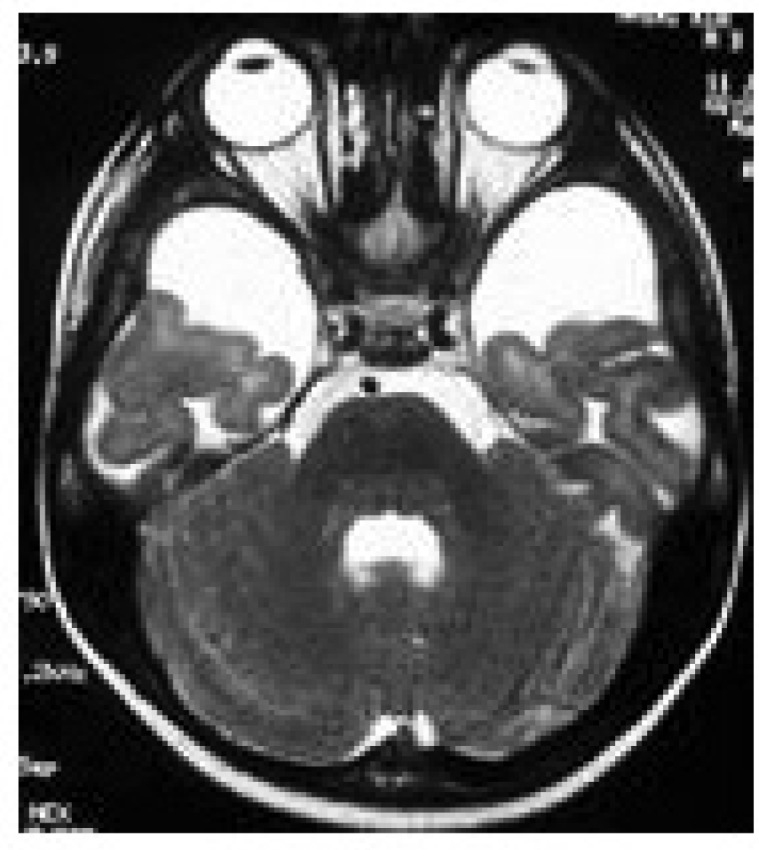
Chronal T2 weighted MRI in patient 7 shows bitemporal arachnoid cysts

Included widened Sylvian fissure in 100%, bitemporal arachnoid cysts in 60%, white matter signal changes in 33% and basal ganglia lesions in 27% of 25 times brain neuroimaging. Neuroimaging studies include16brain CT scan and 9brainsMRI in eleven patients (Figure 2-4).

white matter hyperintensities

**Metabolic analysis finding**

Our patients’ diagnoses were based on blood and/or urine analysis. Bloodglutarylcarnitine and glutarylcarnitine/free carnitine ratio were increased in 11 patients. Urines of 5 patients were tested for organic acid. Increased excretions of glutaric acid and/or 3-hydroxyglutaric acid (organic acids) were reported in these 5 patients.

**GCDH mutation**

We found 7 *GCDH* gene mutations in eleven patients, three of them were not reported before. Five (45%) patients had mutations in exon 7.P 248 L mutation in exon 7 was detected in 3(27%) patients.Three novel mutations were G244C (exon7), A294P (exon8) and N373SMutation in exon10.Four different mutations were seen in 6 Turkish patients. More information about GCDH mutations is shown in Table1, too.

## Discussion

Heterogeneous clinical manifestations, biochemical findings, and GCDH gene mutations are seen in GA1. A severe and treatable inborn error of metabolism usually diagnosed in some countries in neonatal period through expanded newborn screening programusing analysis of glutarylcarnitine in dried blood spots by tandem mass spectrometry(15,16). In areas without newborn screening for acylcarnitine profile such as Iran at present, patients with GA1 usually are diagnosed in symptomatic period or as a selective screening in siblings of a known GA1 patient (17). In most neonates diagnosed withpresymptomatic neonatal period, striatal necrosis and movement disorder can be prevented by low lysine diet and carnitine supplementation (18). An emergency treatment protocol in catabolic states such as fever, gastroenteritis, surgery, immunization, including natural protein stopping, proper hydration, increase carnitine supplementation, appropriate glucose and caloric supply, brisk alkaline urine output, proper antibiotics and antipyretic drugs also are important to prevent neurological sequels(19,20). Acute encephalopathic crisiswas observed as equally as insidious presentation (developmental delay dystonia) in our patients. Acute encephalopathic crisis was reported in majority of patients with GA1 (4) and most patients were presented insidious with developmental delay, macrocephaly (21,22). The discrepancy is due to likely different factors such as the misdiagnosis of initial acute crises by poorly trained physicians or perhaps different GCDH gene mutations.

Enlarge head circumference was described in most neonates with GA1 as the only early sign of this disorder (19).Macrocephaly (occipito-frontal circumference above 95 percentile) was not present at birth in our patients. However, macrocephaly was observed at interview time in our seven (63%) patients.

Dietary management was not continued in five of our severe disabled patients because they did not fill any improvement with this type of therapy. No detectable beneficial effects of long-term dietary treatment were reported in patients with obvious neurological symptoms (6). Although strict low lysine diet is recommended especially in asymptomatic patients up to age of 6 yr old, continuing less strict diet therapy is advised after 6 yr old, too. Carnitine supplementation must be continued lifelong (23). Detection of patients in asymptomatic period and appropriate prospective care reduces clinical and neuroimaging presentation of basal ganglia injury from90% to 35% (19).

Our patients’ neuroimaging findings showed widened Sylvian fissure (bat wing sign) in all patients. Widened Sylvian fissure as characteristic but not pathogenomic neuroimaging finding was reported in 93% of neuroimaging in symptomatic GA1 patients (9).This finding is reported in asymptomatic patients detected on expanded newborn screening suggestive temporal hypoplasia rather than frontotemporal atrophy (10).

White matter abnormalities were detected in 33% of our patients’ brain neuroimaging. White matter changes did not correlate with clinical manifestation. Abnormal white matter changes in Amish patients were reported less frequently (6%) than non-Amish patients (32%). The exact mechanism of this abnormal signal is not understood (19). We found basal ganglia involvement in 27% of neuroimagings of patients. Acute striatal necrosis was reported as the major cause of movement morbidity and mortality in GA1 patients. Nutritional and pharmacological management of newborn detected in expanded newborn screening in asymptomaticperiod-reduced incidence of striatal injury. However, it is unclear why despite early asymptomatic detection of GA1 patients and treatmentintervention; approximately one-third ofthem developed striatal injury (19).

Usually suspected GA1 patients based on clinical, neuroimaging, biochemical findings areconfirmed by molecular or enzymatic study. GCDH gene mutation is used to confirm GA1, carrier detection, genetic consultation and prenatal diagnosis of this disorder. To date, more than 200 mutations in *GCDH* gene have been reported. Some mutations are frequent in specific populations. In Ojibway-Cree linguistic group patients in Manitoba and northwest of Ontario IVS1+5 G > T mutation is prevalent (24). In North Carolina, homozygous mutation E404K (1240 G> A) in exon 10 was reported (25).Selective screening of common gene mutation could be a useful way to decrease time and cost of molecular gene analysis.

In our eleven patients with GA1, seven different*GCDH* gene mutations including three reported previously were detected. Near about half (45%) of *GCDH* mutations occurred in exon7, other mutations occurred in exon8, 10, 11 equally. P 248 L mutation in exon 7 as the most frequent GCDH gene mutation in our study was reported in Turkey and Italy before (13).Other reported mutation in exon 7 was F236 L (26).A 433 V mutation in exon11 was reported from Spain (3). The last mutation T429M in exon11 was reported (27).G244 C in exon7, R294 Q in exon8 and N373 S in exon 10 were novel mutations in this study. *GCDH* gene mutation in patients with GA1 in Khuzestan Province in southeastern of Iran revealed E181Q, S255L, R402G,in order of frequency, were responsible for this disorder in that region (28).R 402 wasthe most common mutation in European patients with GA1 was not detected in our patients, too. More than half of our patients were Turkish and we detect four different mutations in their *GCDH* gene. GA1 is more prevalent in this ethnicity. We did not find any evidence to document genotype and clinical phenotype correlation, owing to high variation of the disease course among patients, as another study (4).

**In Conclusion, **GA1 is a heterogeneous rapid neurodegenerative disorder with available biochemical and genetic test to detect asymptomatic patients. Expanded newborn screening program is recommended in this country. More patients’ genetic analysis is required to know better about *GDCH* gene mutation Iranian patients with GA1. 
